# Multilocus Genotyping of *Giardia duodenalis* Occurring in Korean Native Calves

**DOI:** 10.3390/vetsci8070118

**Published:** 2021-06-23

**Authors:** Sang-Ik Oh, Suk-Han Jung, Han-Kyoung Lee, Changyong Choe, Tai-Young Hur, Kyoung-Min So

**Affiliations:** 1 Division of Animal Diseases & Health, National Institute of Animal Science, Rural Development Administration, Wanju 55365, Korea; ohsangik@korea.kr (S.-I.O.); tnr12gks12@naver.com (S.-H.J.); cychi@korea.kr (C.C.); tyohur@korea.kr (T.-Y.H.); 2 Happy Veterinary Clinic, 289 Nambuk-ro, Gimje 54373, Korea; essenceman@hanmail.net

**Keywords:** *Giardia duodenalis*, assemblage, multilocus genotyping, calves, zoonotic infection

## Abstract

*Giardia duodenalis* is one of the most widely occurring zoonotic protozoan parasites causing diarrheal disease in calves. This study aimed to investigate the prevalence of *G. duodenalis* in Korean native calves and elucidate the causal factors associated with giardiasis in these animals. We investigated the sequences of three genes (*ssu*, *bg*, and *gdh*) of *G. duodenalis* in fecal samples collected from 792 Korean native calves during 2019–2020. Data were analyzed with regard to age, sex, sampling season, and the fecal sample type (based on its physical characteristics). The samples were screened for the three genes mentioned above, and 44 samples (5.6%) were *G. duodenalis*-positive. Polymerase chain reaction results showed a significantly higher prevalence of the infection in calves aged ≥1 month and in those with watery diarrhea in spring season. Screening for the gene sequences *ssu* (87.5%), *bg* (96.2%), and *gdh* (96.7%) revealed that most of the *G. duodenalis*-positive samples belonged to assemblage E. Four of the *G. duodenalis*-positive samples belonged to the zoonotic assemblage A. This study highlights the importance of continuous surveillance of genetic mutations in *G. duodenalis* for the detection of emerging variants of zoonotic *G. duodenalis* in calves.

## 1. Introduction

*Giardia duodenalis* (synonyms *Giardia intestinalis*, *Giardia lamblia*) is a zoonotic protozoan parasite that causes intestinal diseases in various host species. Infection occurs following ingestion of infective stages of this pathogen (cysts) via the fecal–oral route or drinking contaminated water [1,2,3]. Giardiasis in cattle is caused by *G. duodenalis* and is one of the most important parasitic diseases associated with diarrhea in calves [4]. Although the pathogenesis of this protozoan parasite is not fully understood yet, the pathogen is known to cause diffuse shortening of brush border microvilli and malabsorption of water, sodium, and glucose [5]. Consequently, cattle infected with *G. duodenalis* show clinical signs such as malabsorptive diarrhea and reduced weight gain [5,6]. *G. duodenalis* has a broad host range, including humans, cattle, sheep, goats, pigs, dogs, cats, and rats, and is thus a potential threat to public health [7,8]. Previous studies have reported that cattle are one of the important hosts responsible for zoonotic transmission of giardiasis to humans, and molecular epidemiological analysis of *G. duodenalis* from cattle is required to elucidate the role of cattle as a potential zoonotic reservoir [9].

Recent studies have reported that *G. duodenalis* consists of eight distinct assemblages (A to H), which appear to have different host ranges [7,10]. Among these assemblages, A and B are infective in humans and many other animals, including ruminants, dogs, rodents, and marine mammals [2,7], whereas other assemblages are mostly host-specific; assemblage E is infective in ruminants and pigs, C and D in dogs, and G in rodents [7]. Thus far, assemblages A, B, and E have been detected in cattle [7,8]. Interestingly, previous studies suggested that calves are more frequently infected with zoonotic assemblages A and B than adult cattle are [6,11].

According to previous studies, the prevalence of *G. duodenalis* was 13.1% (2013–2015) and 10.0% (2017) in Korean native calves with diarrhea and normal feces, respectively [12,13]. These studies also reported that all the analyzed fecal samples from diarrheic calves in Korea in 2013–2015 belonged to assemblage E [12], and 2.2% of those analyzed from normal fecal samples in 2017 belonged to assemblage A [13]. These two studies indicated that *G. duodenalis* is a major concern of infection in Korean native cattle farms, and zoonotic *G. duodenalis* (assemblage A) could have emerged in Korea in 2017. Therefore, it is essential to continuously monitor by testing fecal samples from calves and adult cattle to determine the prevalence and identify the genotypes of *G. duodenalis* responsible for causing zoonotic infections.

Most of the previous studies conducted in Korea have identified *G. duodenalis* in calves by a single gene locus [12,13]. Therefore, in this study, we broadened the scope by performing a large-scale analysis of *G. duodenalis*, with multilocus genotyping based on sequences of the small subunit (SSU) rRNA, β-giardin, and glutamate dehydrogenase (GDH). We also analyzed the factors associated with the occurrence of *G. duodenalis*, including age and sex of calves, sampling season, and the fecal sample type (based on its physical characteristics).

## 2. Materials and Methods

### 2.1. Sample Collection

**Figure 1:**
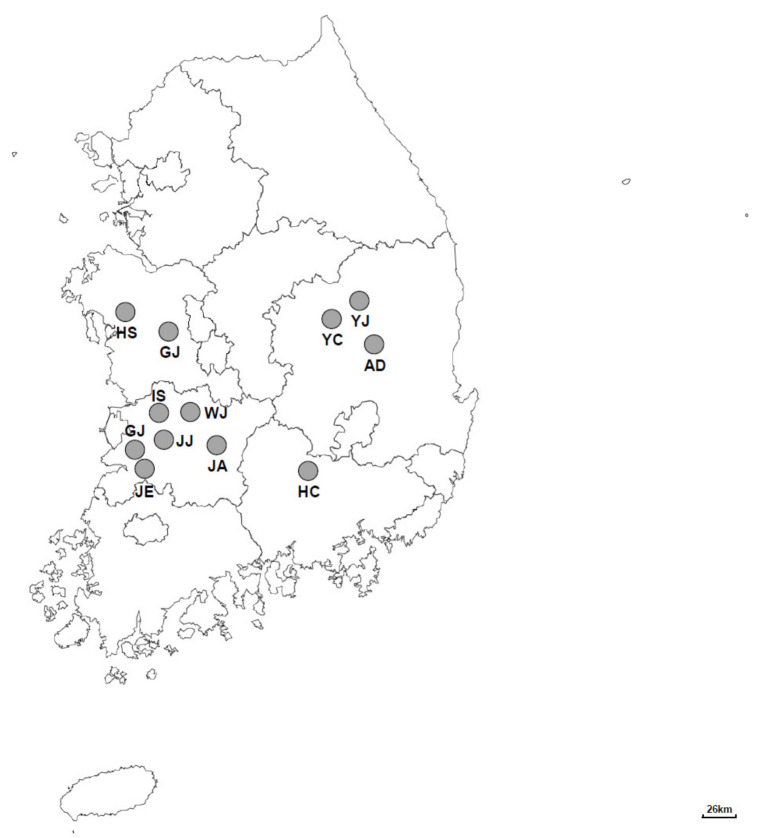
A map of the Republic of Korea showing the location of the sampling sites from where fecal samples were collected from Korean native calves. GJ: Gimje (*n* = 624); JE: Jeongeup (*n* = 97); YJ: Youngju (*n* = 19); IS: Iksan (*n* = 14); GJ: Gongju (*n* = 13); HS: Hongseong (*n* = 7); HC: Hapcheon (*n* = 6); JJ: Jeonju (*n* = 3); AD: Andong (*n* = 3); WJ: Wanju (*n* = 2); JA: Jinan (*n* = 2); YC: Yecheon (*n* = 2).

A total of 792 fecal samples were obtained from 291 Korean native cattle farms between May 2019 and September 2020. These cattle farms were located in 12 different regions (Gimje, Jeongeup, Youngju, Iksan, Gongju, Hongseong, Hapcheon, Jeonju, Andong, Wanju, Jinan, and Yecheon) in the central region of the Republic of Korea (Figure 1). The details of each calf were recorded, including geographical location, age, sex, and the sample collection date. The type of feces was also recorded and categorized as normal, pasty, watery, or hemorrhagic according to its physical characteristics. Fresh feces from calves were collected in 50-milliliter stool specimen collection tubes immediately after defecation, using polyethylene gloves. All the samples were packed in boxes with icepacks and sent to the laboratory. Feces were stored at 4 °C until the extraction of genomic DNA. 

### 2.2. DNA Extraction and Polymerase Chain Reaction (PCR) Amplification

Genomic DNA was extracted from fecal samples using the QIAamp fast DNA Stool Mini Kit (QIAgen, Hilden, Germany) according to the manufacturer’s instructions. To amplify the SSU rRNA gene (*ssu*) and β-giardin gene (*bg*) fragments from *G. duodenalis*, nested PCR was conducted for each gene using primer sets described in a previous study [14]. For amplification of the GDH gene (*gdh*) fragments, semi-nested PCR was performed as reported previously [14].

### 2.3. Sequencing and Phylogenetic Analysis

All the fecal samples identified as *G. duodenalis*-positive by PCR were used for sequencing. Amplicons were sequenced directly in both directions using the Bigdye Terminator Cycle Sequencing Ready Reaction Kit V.3.1 (Applied Biosystems, Foster City, CA, USA) on the 3730XL Capillary DNA sequencer machine (Applied Biosystems) by SolGent Co., Ltd. (Daejeon, Korea). If the sequencing result was unclear, the experiment was repeated until a clear result was obtained. The obtained sequences of *ssu*, *bg*, and *gdh* were identified from the public data libraries of the National Center for Biotechnology Information (NCBI, USA) using the basic local alignment search tool (BLAST) and then aligned using Clustal Omega Alignment Tools (http://www.ebi.ac.uk/tools/msa/clustalo/, accessed on 9 April 2021). The nucleotide sequences of the *G. duodenalis*-positive samples were deposited in the NCBI GenBank database: MW876381–MW876412 for the SSU rRNA locus, MW876413–MW876438 for the β-giardin locus, and MW876439–MW876467 for the GDH locus. For phylogenetic analysis, we collected the nucleotide sequences of *G. duodenalis* variants from various host species in various countries from the NCBI GenBank database. Phylogenetic trees were constructed using the neighbor-joining method. The sequence data were sampled using 1000 replicates for bootstrap analysis using the Mega-X software (version 10.0.4) with the Kimura two-parameter option.

### 2.4. Statistical Analysis

A binominal logistic regression analysis was used to compare the prevalence of *G. duodenalis* by calf age and sex, sampling season, and fecal sample type. The variables were grouped as follows: six age groups (≤2, 3–4, 5–6, 7–8, 9–12, and >12 weeks), three groups for sex (male, female, and unknown), four groups for sampling season (spring, summer, autumn, and winter), and four fecal types (normal, pasty, watery, and hemorrhagic feces). The level of significance was set at *p* < 0.05. The odds ratio (OR) with 95% confidence intervals (CI) was estimated to show the strength of associations. Statistical analysis was performed using SPSS version 26.0 (IBM, Armonk, NY, USA). In addition, the Kappa (κ) values, which range from 1 to 0, were interpreted according to a previous study [15], where <0: no agreement; 0–0.20: slight agreement; 0.21–0.40: fair agreement; 0.41–0.60: moderate agreement; 0.61–0.80: substantial agreement; and 0.81–1.00: almost perfect agreement.

## 3. Results

### 3.1. Prevalence and Risk Factors of G. duodenalis

The prevalence of *G. duodenalis* was 5.6% (*n* = 44) among 792 calves from 37 farms (12.7% of 271 tested farms), as determined by PCR (Table 1). *G. duodenalis* was most prevalent in calves aged 7–8 weeks (15.4% of 26 calves) and ≥12 weeks (15.4% of 13 calves), followed by those aged 9–12 weeks (15.2% of 46 calves) and 5–6 weeks (14.0% of 107 calves). The calves aged less than 1 month showed a lower prevalence of *G. duodenalis* compared with those aged more than 1 month. There was no significant difference between the occurrence of *G. duodenalis* in male (5.7%) and female (5.6%) calves. With respect to seasons, the prevalence of *G. duodenalis* was 6.7% in spring (March–May), 6.0% in summer (June–August), 7.4% in autumn (September–November), and 1.5% in winter (December–February). Furthermore, *G. duodenalis* was frequently detected in hemorrhagic (12.5%) and watery (9.8%) diarrheal feces, but rarely detected in pasty (2.9%) and normal (2.3%) feces. Based on the statistical analysis, there was significantly lower prevalence in calves under 2 weeks of age (OR = 0.084, *p* = 0.006) than in those over 2 weeks old. In addition, *G. duodenalis* was significantly more prevalent in watery feces (OR = 4.319, *p* = 0.001) and in fecal samples from the spring (OR = 6.446, *p* = 0.018).

### 3.2. Detection of G. duodenalis by the Amplification of ssu, gdh, and bg

**Figure 2:**
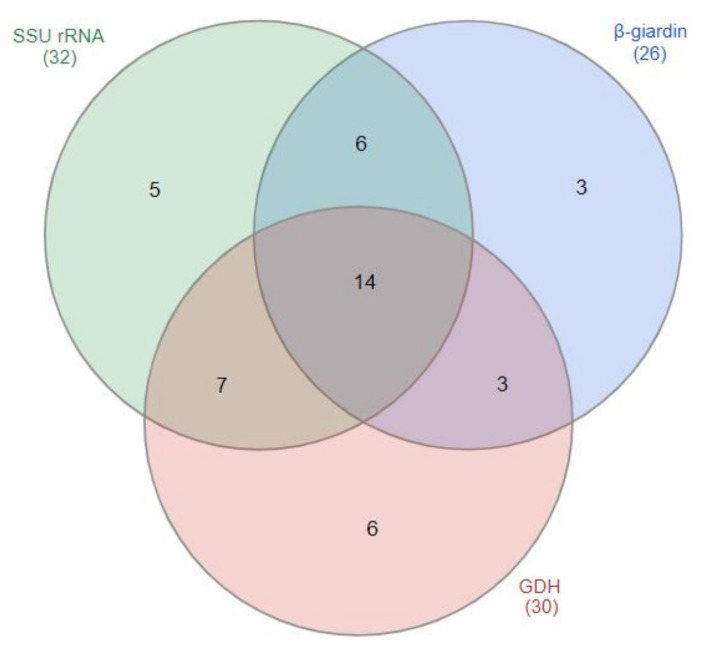
Venn diagram of *Giardia-duodenalis*-positive samples (*n* = 44). Numbers of fecal samples that were PCR positive for *ssu* rRNA gene and/or *bg* gene and/or *gdh* gene are provided.

Among the 44 *G.* *duodenalis*-positive fecal samples, 32 fecal samples (72.7%) were *ssu*-positive, 26 samples (59.1%) were *bg*-positive, and 30 samples (68.2%) were *gdh*-positive (Figure 2). Of the 32 *ssu* rRNA gene-positive samples, 62.5% (*n* = 20) were *bg*-positive and 65.6% (*n* = 21) were *gdh*-positive. Only 31.8% (*n* = 14) of samples tested positive for all three genes. The κ-values of 0.68 and 0.66 suggest a good agreement between *ssu*- and *bg*-positive samples and *ssu-* and *gdh-*positive samples, respectively. A moderate agreement was observed between *bg*- and *gdh*-positive samples (κ = 0.59).

### 3.3. Nucleotide Sequencing

A total of 28 sequences were identical among the 32 *ssu*-positive sequences, three were different from the 28 sequences but identical to each other, and one sequence showed a marginal difference from the other sequences. Eight and six sequences were identified in *bg* and *gdh* sequences, respectively (Table 2 and Table 3). All the sequences were deposited in the NCBI GenBank database under the respective accession numbers.

### 3.4. Phylogenetic Analysis

**Figure 3:**
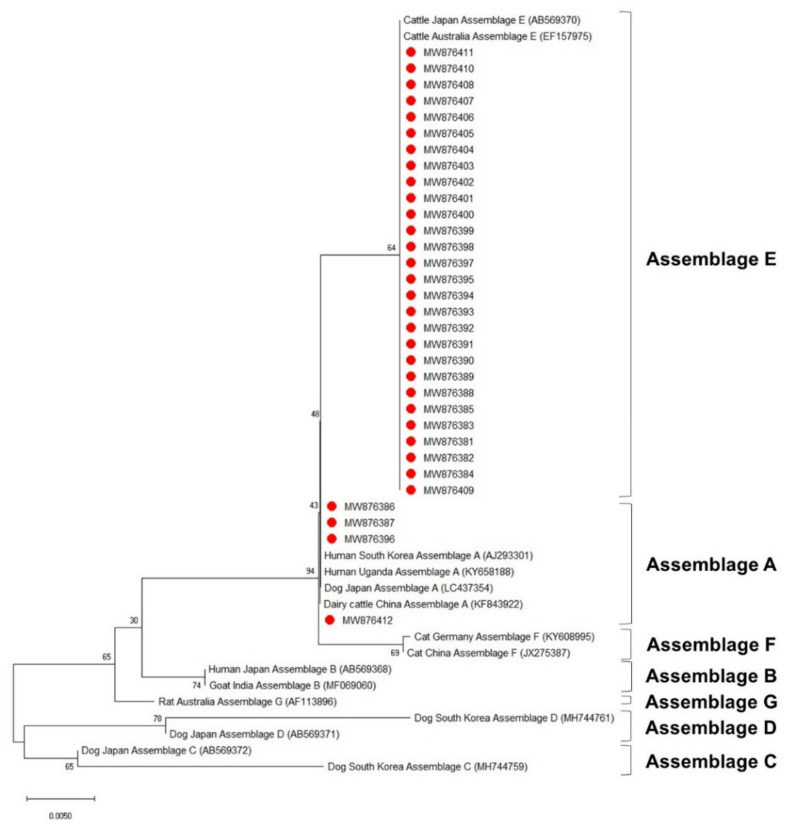
Phylogenetic tree of *G. duodenalis* based on SSU rRNA gene sequences. The neighbor-joining method was used to construct the tree; the red circles indicate the sequences detected in this study. The GenBank accession numbers are shown in parentheses, and the *G. duodenalis* assemblages are indicated with one-sided square brackets. Tree reliability was tested by running 1000 bootstrap replicates.

**Figure 4:**
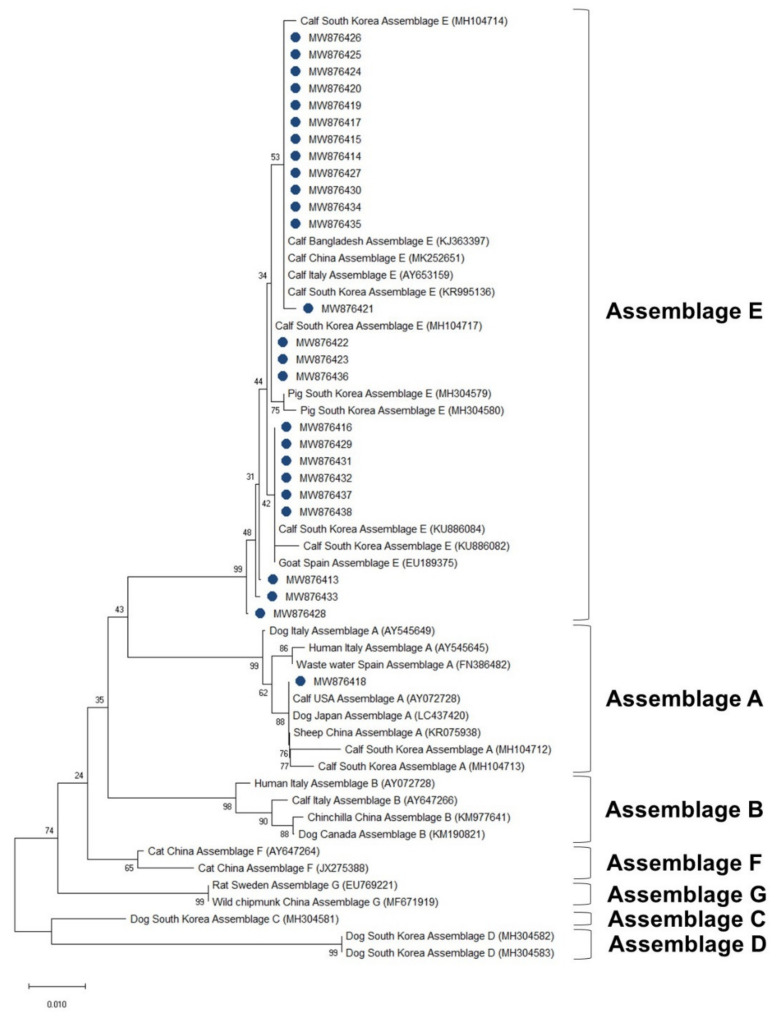
Phylogenetic tree of *G. duodenalis* based on β-giardin gene sequences. The neighbor-joining method was used to construct the tree; the blue circles indicate the sequences detected in this study. The GenBank accession numbers are shown in parentheses, and the *G. duodenalis* assemblages are indicated with one-sided square brackets. Tree reliability was tested by running 1000 bootstrap replicates.

**Figure 5:**
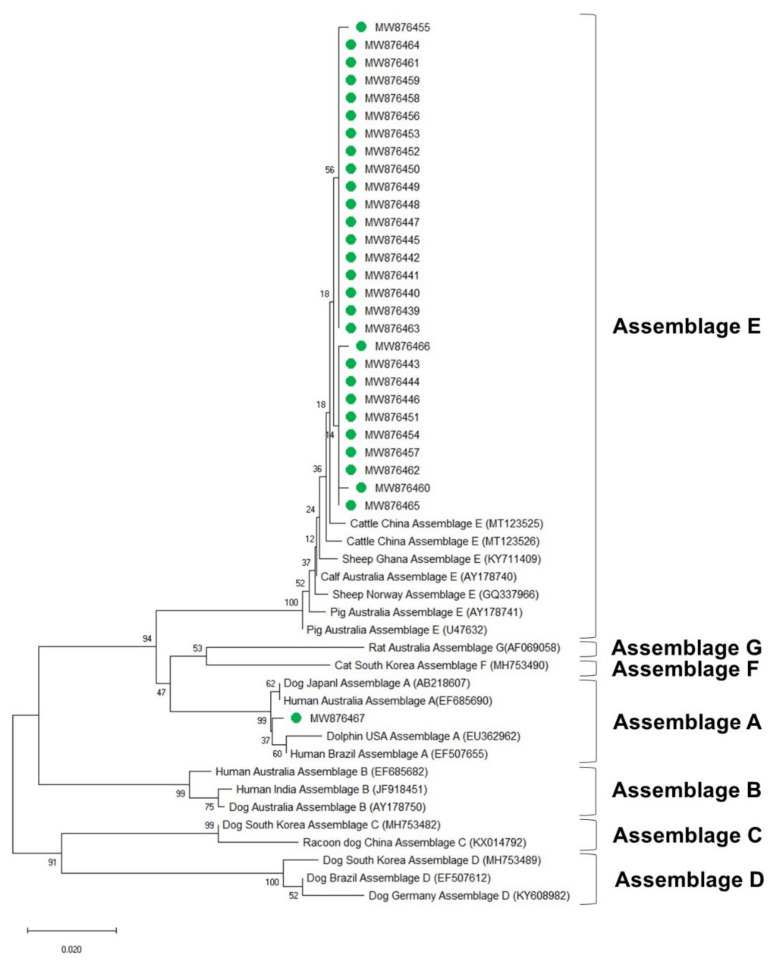
Phylogenetic tree of *G. duodenalis* based on GDH gene sequences. The neighbor-joining method was used to construct the tree; the green circles indicate the sequences detected in this study. The GenBank accession numbers are shown in parentheses, and the *G. duodenalis* assemblages are indicated with one-sided square brackets. Tree reliability was tested by running 1000 bootstrap replicates.

The phylogenetic analysis results of the *ssu*, *bg*, and *gdh* sequences are shown in Figure 3, Figure 4 and Figure 5, respectively. Most of the *ssu*-positive *G. duodenalis* samples from Korean native calves belonged to assemblage E (87.5%, *n* = 28). Four of the *ssu-*positive samples (12.5%) belonged to assemblage A (Figure 3). A total of 25 *bg*-positive samples (96.2%) belonged to assemblage E, and only one sample (3.8%) belonged to assemblage A (Figure 4). All *gdh*-positive fecal samples (96.7%) belonged to assemblage E except one (3.3%), which belonged to assemblage A (Figure 5).

## 4. Discussion

The present study (2019–2020) indicated that 5.6% of the tested Korean native calves were infected with *G. duodenalis*, which is lower than the number of calves reportedly infected in 2013–2015 (13.1%, *n* = 590) and 2017 (10.0%, *n* = 90) [12,13]. The rate of infection (5.6%) observed in this study was also lower than that reported from other countries, including the United States (44%), Belgium (31.3%), Egypt (13.3%), Vietnam (10.2%), China (9.2%), and Thailand (6.0%) [16,17,18,19,20,21]. These differences may be ascribed to the fact that the determinants of infection rates are varied and affected by many factors, including the geoecological conditions, diagnostic methods, sample size, sample collection period, and age of the sampled animals [6,12,22]. Therefore, the current study investigated the influential factors behind the occurrence of *G. duodenalis* infection in Korean native calves.

As shown in Table 1, the neonatal calves (≤2 weeks old) were associated (OR = 0.084, *p* = 0.006) with prevalence of *G. duodenalis* in feces. *G. duodenalis* was more prevalent in calves aged ≥1 month (14.6%) than in those aged <1 month (2.7%). This result was in line with the findings of previous studies in China in 2016 and 2020, which reported the highest prevalence of *G. duodenalis* infection in calves aged 1–3 months (19.6% and 10.7%, respectively) [19,23]. Thus, given that neonatal calf diarrhea commonly occurs in calves at around 2 weeks of age, *G. duodenalis* may not be the main pathogen responsible for this disease in these animals [24]. However, there is a need to continuously monitor this protozoan parasite to prevent outbreaks of giardiasis in calf-breeding farms. In the present study, no significant difference was observed in the prevalence of the infection between male (5.7%, *p* = 0.998) and female (5.6%, *p* = 0.161) calves, which was consistent with the findings of a previous study in Korea [25]. Furthermore, the prevalence of *G. duodenalis* infection was the highest in autumn (7.4%, *n* = 6 out of 81), followed by spring (6.7%, *n* = 17 out of 256), summer (6.0%, *n =* 19 out of 320), and winter (2.7%, *n* = 2 out of 135). Among the four seasons, the fecal samples of calves from the spring were significantly (OR = 6.446, *p* = 0.018) associated with the presence of *G. duodenalis*. The current findings are consistent with the findings of previous studies in Korea [12] and China [23], in which *G. duodenalis* infection was most frequently detected in the warm season. With regard to fecal sample type, the occurrence rate of *G. duodenalis* was the highest in calves with hemorrhagic feces (12.5%), followed by watery diarrheal (9.8%), pasty (2.9%), and normal (2.3%) feces. According to the binominal logistic regression model analysis, the watery fecal samples were significantly (OR = 4.319, *p* = 0.001) associated with the occurrence of *G. duodenalis*. This result is in accordance with the findings of a previous study in Korea, which reported a statistical association between *G. duodenalis* detection and the type of fecal sample [12]. However, other studies reported no significant association between the fecal sample type and *G. duodenalis* infection [13,26]. These overall results suggest that veterinarians and farm workers should suspect *G. duodenalis* infection in calves (≥1 month of age) with watery diarrhea in the spring. Moreover, hemorrhagic diarrhea could also be a result of co-infection with *G. duodenalis*, and thus, the calves must be screened for other pathogenic infections that could cause hemorrhagic diarrhea.

The results of this study highlight that there is no perfect PCR primer set to detect all *G. duodenalis*-positive samples at once. Among the three PCR primer sets used in this study, the SSU rRNA gene primer set was more effective in detecting *G. duodenalis* than the protein-coding gene (β-giardin and GDH) primer sets. This finding is in agreement with previous studies [27,28]. Although the three PCR primer sets (*ssu*, *bg*, and *gdh*) showed a markedly good agreement with each other according to their κ-values, laboratory PCR diagnosis of *G. duodenalis* should be conducted using different primers for detecting all infected calves. Further studies are needed to establish a PCR diagnostic standard that can determine *G. duodenalis* infection with high sensitivity and specificity.

*G. duodenalis* exhibits host-specific characteristics that vary depending on their assemblages [4,10]. The phylogenetic analysis in this study suggested that the SSU rRNA gene is strongly conserved among *G. duodenalis* assemblages. The *ssu* sequence analysis revealed that four sequences (MW876386, MW876387, MW876396, and MW876412) belong to zoonotic assemblage A, whereas *bg* and *gdh* had only one sequence each belonging to assemblage A (MW876418 and MW876467, respectively). The *bg* (MW876418) and *gdh* sequences (MW876467) belonging to assemblage A were derived from two samples that also contained two *ssu* sequences belonging to assemblage A (MW876386 and MW876412). These different phylogenetic results according to the sequences of each locus suggest that assigning one locus is insufficient for laboratory diagnosis and identification of the assemblage of *G. duodenalis*.

In assemblage E, we found only one sequence at the *ssu* locus but seven at the *bg* locus and five at the *gdh* locus. The gene *bg* showed the greatest variability, followed by *gdh*, then *ssu*. The results implied that *bg* is the most feasible target for determination of the genetic diversity of *G. duodenalis*. A total of seven different sequences were identified from the *bg* gene sequence of assemblage E, three of which were novel types compared with a previous study on calves in Korea [12]. This result suggests the occurrence of genetic mutations in assemblage E of *G. duodenalis* infecting calves in Korea. Therefore, continuous monitoring is needed to detect emerging novel types of *G. duodenalis* and obtain updated genetic information to prevent the outbreak of infections caused by a novel *G. duodenalis* strain. Moreover, many recent studies commonly performed multilocus genotyping with three genes, namely *bg*, *gdh*, and triose phosphate isomerase (*tpi*), which could identify multilocus genotypes (MLGs) of *G. duodenalis* [19,23]. Thus, further experiments with *tpi* sequencing are needed to reveal the MLGs of *G. duodenalis* from Korean native calves. The prevalence of *G. duodenalis* belonging to assemblage A (9.1%) was lower in this study than that observed in previous studies in China, the USA, Belgium, and Portugal [16,17,22,29]. According to the results of the present study, the four calves that harbored zoonotic *G. duodenalis* (belonging to assemblage A) were all aged 1–2 months and had watery diarrhea. These results are in contrast to the findings of a previous study, which showed that *G. duodenalis* assemblage A was identified in calves aged 1 month with normal fecal discharge [13]. Furthermore, despite the small number of fecal samples collected in autumn, most of the *G. duodenalis*-positive samples (75%) belonging to assemblage A were detected in this season. These results are similar to the findings of a previous study, which reported that all *G. duodenalis*-positive samples belonging to assemblage A were isolated in autumn [30]. As *G. duodenalis* belonging to assemblage A is not host-specific, some vectors with increased activity in autumn (e.g., humans, wild rodents, or dogs), may play an important role in pathogen transmission. Therefore, further studies are needed to identify the potential vectors for zoonotic transmission of *G. duodenalis* from cattle farms. The present findings provide valuable information for similar monitoring studies in the future.

## 5. Conclusions

This study provides updated data regarding the prevalence of and the factors associated with *G. duodenalis* infection in Korean native calves. The prevalence of *G. duodenalis* infection was 5.7% (12.7% at the farm level) in this study. Calves aged ≥1 month or producing watery feces in the spring should be carefully monitored by veterinarians to prevent *G. duodenalis* transmission to healthy calves. In addition, this is the first multilocus genotyping study of *G. duodenalis* from the feces of native calves in Korea. The results highlight that analysis of the *ssu* gene sequence is an effective tool to detect the presence of *G. duodenalis* in fecal samples and determine their assemblages. The *bg* gene is useful to investigate the genetic diversity in *G. duodenalis*. A total of four out of 792 samples (0.5%) belonged to assemblage A as identified by *ssu* sequencing and were potential zoonotic pathogens. The present findings could help to understand the genetic relationships between *G. duodenalis* variants distributed across Korea.

## Figures and Tables

**Table 1:** Detection of *Giardia duodenalis* by PCR in the feces of 792 Korean native calves.

Classification		No. Tested	PCR			
			No. of Positive Samples (%)	95% CI *	OR *	*p*-Value
Constant	-	-	-	-	0.06	<0.001
Age	≤2 weeks **	462	7 (1.5)	0.4–2.6	0.084	0.006
	3–4 weeks	138	9 (6.5)	2.4–10.6	0.471	0.399
	5–6 weeks	107	15 (14.0)	7.4–20.6	1.081	0.929
	7–8 weeks	26	4 (15.4)	1.5–29.3	1.045	0.965
	9–12 weeks	46	7 (15.2)	4.8–25.6	1.072	0.940
	≥12 weeks	13	2 (15.4)	0–35.0	-	-
Sex	Male	402	23 (5.7)	3.5–8.0	<0.001	0.998
	Female	373	21 (5.6)	3.3–8.0	0.618	0.161
	Unknown	17	-	-	-	-
Season	Spring **	256	17 (6.7)	3.6–9.7	6.446	0.018
	Summer	320	19 (6.0)	3.4–8.5	3.393	0.113
	Autumn	81	6 (7.4)	1.7–13.1	5.016	0.062
	Winter	135	2 (1.5)	0–3.5	-	-
Fecal type	Normal	349	8 (2.3)	0.7–3.9	-	-
	Pasty	137	4 (2.9)	0.1–5.7	1.332	0.654
	Watery **	234	23 (9.8)	6.0–13.6	4.319	0.001
	Hemorrhagic	72	9 (12.5)	4.9–20.1	2.123	0.169
Total		792	44 (5.6)	4.0–7.2	-	-

* OR: Odds ratio, CI: Confidence interval. ** *p* < 0.05.

**Table 2:** Nucleotide variations among the 26 β-giardin sequences detected from the feces of 792 calves.

Accession No. ^1^	Nucleotides at Variable Positions			
	45	150	252	393
MW876414, 15, 17, 19, 20, 24–27, 30, 34, 35	T	A	T	T
MW876416, 29, 31, 32, 37, 38	C	A	C	T
MW876422, 23, 36	T	A	C	T
MW876413	T	A	C	C
MW876421	T	G	T	T
MW876428	T	G	C	C
MW876433	C	A	C	C

**^1^** MW876418 was marginally different from the other 25 β-giardin sequences.

**Table 3:** Nucleotide variations among the 30 GDH sequences detected from the feces of 792 calves.

Accession No. ^1^	Nucleotides at Variable Positions			
	258	306	342	387
MW876439–42, 45, 47–50, 52, 53, 56, 58, 59, 61, 63, 64	C	T	A	C
MW876443, 44, 46, 51, 54, 57, 62, 65	C	C	A	C
MW876455	A	T	A	C
MW876460	C	C	G	C
MW876466	C	C	A	T

**^1^** MW876467 was marginally different from the other 29 GDH sequences.

## Data Availability

Not applicable.
